# Characterization of Four Multidrug Resistance Plasmids Captured from the Sediments of an Urban Coastal Wetland

**DOI:** 10.3389/fmicb.2017.01922

**Published:** 2017-10-10

**Authors:** Ryan T. Botts, Brooke A. Apffel, C. J. Walters, Kelly E. Davidson, Ryan S. Echols, Michael R. Geiger, Victoria L. Guzman, Victoria S. Haase, Michal A. Montana, Chip A. La Chat, Jenna A. Mielke, Kelly L. Mullen, Cierra C. Virtue, Celeste J. Brown, Eva M. Top, David E. Cummings

**Affiliations:** ^1^Department of Mathematical, Information and Computer Sciences, Point Loma Nazarene University, San Diego, CA, United States; ^2^Department of Biology, Point Loma Nazarene University, San Diego, CA, United States; ^3^Department of Biological Sciences, Institute for Bioinformatics and Evolutionary Studies, University of Idaho, Moscow, ID, United States

**Keywords:** antibiotic resistance genes, plasmids, wetlands, mobile genetic elements, horizontal gene transfer, conjugation, self-transmissible, mobilizable

## Abstract

Self-transmissible and mobilizable plasmids contribute to the emergence and spread of multidrug-resistant bacteria by enabling the horizontal transfer of acquired antibiotic resistance. The objective of this study was to capture and characterize self-transmissible and mobilizable resistance plasmids from a coastal wetland impacted by urban stormwater runoff and human wastewater during the rainy season. Four plasmids were captured, two self-transmissible and two mobilizable, using both mating and enrichment approaches. Plasmid genomes, sequenced with either Illumina or PacBio platforms, revealed representatives of incompatibility groups IncP-6, IncR, IncN3, and IncF. The plasmids ranged in size from 36 to 144 kb and encoded known resistance genes for most of the major classes of antibiotics used to treat Gram-negative infections (tetracyclines, sulfonamides, β-lactams, fluoroquinolones, aminoglycosides, and amphenicols). The mobilizable IncP-6 plasmid pLNU-11 was discovered in a strain of *Citrobacter freundii* enriched from the wetland sediments with tetracycline and nalidixic acid, and encodes a novel AmpC-like β-lactamase (*bla*_*WDC*-1_), which shares less than 62% amino acid sequence identity with the PDC class of β-lactamases found in *Pseudomonas aeruginosa*. Although the IncR plasmid pTRE-1611 was captured by mating wetland bacteria with *P. putida* KT2440 as recipient, it was found to be mobilizable rather than self-transmissible. Two self-transmissible multidrug-resistance plasmids were also captured: the small (48 kb) IncN3 plasmid pTRE-131 was captured by mating wetland bacteria with *Escherichia coli* HY842 where it is seemed to be maintained at nearly 240 copies per cell, while the large (144 kb) IncF plasmid pTRE-2011, which was isolated from a cefotaxime-resistant environmental strain of *E. coli* ST744, exists at just a single copy per cell. Furthermore, pTRE-2011 bears the globally epidemic *bla*_*CTX-M*-55_ extended-spectrum β-lactamase downstream of IS*Ecp1*. Our results indicate that urban coastal wetlands are reservoirs of diverse self-transmissible and mobilizable plasmids of relevance to human health.

## Introduction

Bacterial resistance to antibiotics is ancient (D’Costa et al., 2011) and pre-dates their clinical use to prevent and cure bacterial infections (Allen et al., 2009; Lang et al., 2010). However, since the beginning of the current “antibiotic era” less than a 100 years ago, human-induced selective pressures against antibiotic-susceptible strains has led to an intractable rise in the frequency of resistant populations (e.g., Bonnet, 2004; Falagas et al., 2005; Giske et al., 2008; Strahilevitz et al., 2009; Nordmann et al., 2011). Global public health is now facing an epidemic of bacterial infections with reduced susceptibility to front-line clinical antibiotics such as cephalosporins and fluoroquinolones (Levy and Marshall, 2004), leading to therapeutic failure, worsening patient outcomes, and increased financial burdens on individuals and the health care system as a whole (Centers for Disease Control and Prevention [CDC], 2013; O’Neill Commission, 2014; World Health Organization [WHO], 2014). Viable solutions are urgently needed to protect the long-term efficacy of the antimicrobial agents that we have come to rely upon so heavily.

Chromosomal mutations in genes coding for antibiotic targets can provide protection from the action of an antibacterial drug. However, such mutations can only be passed on to the next generation of bacteria in a vertical manner, from parent to offspring, limiting the rate and extent of the spread of the resistance phenotype. By contrast, horizontal transfer of resistance genes via plasmids allows for the spread of resistance elements to other bacteria in the same generation, creating opportunity for rapid and extensive spread of the resistance phenotype. Plasmids are extra-chromosomal genetic elements that are capable of translocation from one host cell to another, sometimes across broadly divergent phylogenetic boundaries (Courvalin, 1994). Many plasmids encode the necessary machinery to transfer by conjugation; these are known as self-transmissible plasmids (see for example Francia et al., 2004; Smillie et al., 2010). Others, the mobilizable plasmids, can only transfer between cells with the help of a self-transmissible plasmid. Much of the recent rise in antibiotic resistance is very likely due to the rapid spread of resistance plasmids by horizontal gene transfer (Subbiah et al., 2011; Zacharczuk et al., 2011; Cantón et al., 2012; Hiroi et al., 2012).

While the responsible use of antibiotics to prevent and treat infections in humans and veterinary medicine plays an important role in selecting for this increased resistance (e.g., Hsu et al., 2010), many also argue that their use in applications other than bacterial infections (e.g., growth promotion in food animals, human viral illnesses, etc.) may be contributing to the problem (Levy, 2002; Smith et al., 2002; Shea, 2003; Salyers and Whitt, 2005). Furthermore, there is a growing concern that the release of antibiotics, antibiotic-resistant bacteria, and antibiotic resistance genes into natural habitats may be exacerbating the clinical situation (Pruden et al., 2006; American Academy of Microbiology, 2009; Knapp et al., 2010).

Previous work on the fate of antibiotic-resistant bacteria and their resistance genes in the natural environment has focused largely on the impacts of animal production facilities (e.g., Burgos et al., 2005; Koike et al., 2007; Li et al., 2011; Brauer et al., 2016), which employ antibiotics for disease prevention and treatment as well as growth promotion. There is growing evidence, however, that bacteria of the human colon carry numerous acquired resistance genes (Salyers et al., 2004; Monira et al., 2017). It is not surprising, then, that human sewage has been documented as a vast reservoir of acquired resistance genes (Bönemann et al., 2006; Schlüter et al., 2007; Szczepanowski et al., 2009), many of which are encoded on self-transmissible plasmids. Metagenomic studies suggest that waste water carries a tremendous diversity of novel plasmids that have the potential to recombine with one another, creating raw material upon which natural selection may act (Sentchilo et al., 2013). Release of these genes into the natural environment via sewage has been reported by our lab (Cummings et al., 2011; Borgogna et al., 2016) and others (Novais et al., 2005; Zhang et al., 2009).

Given the relative ease by which prokaryotes exchange genetic information, even in the absence of an obvious selective pressure (Salyers and Amábile-Cuevas, 1997; Lili et al., 2007), the release of plasmid-bound resistance genes into natural environments may pose a real public and environmental health risk. Most acquired resistance genes likely evolved in natural habitats before transferring into human pathogens through various horizontal gene transfer mechanisms (Martínez, 2009). Many of the pentapeptide repeat proteins involved in quinolone resistance, for example, have been shown to derive from chromosomal genes in marine bacteria (Poirel et al., 2005a,b). Similarly, *Shewanella* spp., common in coastal wetlands, have been identified as rich sources of integrons, β-lactamases, and sulfonamide resistance genes (Poirel et al., 2004; Ramírez et al., 2010). To understand the trajectories by which antibiotic resistance genes spread into clinically relevant bacteria, we have focused our attention on the plasmid reservoirs that carry them.

The aim of this study was to capture and characterize resistance plasmids from an urban wetland receiving mixed runoff that intermittently includes untreated human waste water. We describe the complete nucleotide sequences of four novel plasmids from this environment and the antibiotics to which they confer resistance.

## Materials and Methods

### Description of the Study Site

The Tijuana River Estuary (TRE) (N32°33′, W117°07′) is a 10-km^2^ National Estuarine Research Reserve, National Wildlife Refuge, and Wetland of International Importance (Ramsar site #1452) on the United States-Mexico border in Imperial Beach, California, United States (Zedler et al., 1992). The 4400-km^2^ watershed includes urban areas, agricultural land, and open chaparral and sage scrub. The South Bay International Waste Water Treatment Plant, which treats 25 million gallons of raw sewage per day from Tijuana, Mexico, is situated immediately upstream of the estuary, where it releases untreated municipal waste water into the river and wetlands during periods of heavy rain (Conway et al., 1985). Surface sediments (50 g from top 5 mm) were collected with sterile spatulas after rainfall and transported to the laboratory in sterile centrifuge tubes on ice.

### Antibiotic Abbreviations

AM, ampicillin; AN, amikacin; ATM, aztreonam; C, chloramphenicol; CAZ, ceftazidime; CF, cefalothin; CIP, ciprofloxacin; CL, colistin; CTX, cefotaxime; CXM, cefuroxime; D, doxycycline; ETP, ertapenem; FEP, cefepime; FOX, cefoxitin; GAT, gatifloxacin; IPM, imipenem; K, kanamycin; LOM, lomefloxacin; LVX, levofloxacin; MEM, meropenem; MXF, moxifloxacin; NA, nalidixic acid; NOR, norfloxacin; OFX, ofloxacin; PIP, piperacillin; RIF, rifampicin; S, streptomycin; SAM, ampicillin/sulbactam; SPX, sparfloxacin; SXT, sulfamethoxazole/trimethoprim; TE, tetracycline; TIC, ticarcillin; TIM, ticarcillin/clavulanic acid; TZP, piperacillin/tazobactam; ZEO, Zeocin.

### Endogenous Plasmid Capture

In the laboratory, 50 g surface sediments were homogenized by hand. A 1-g sub-sample was added to Luria-Bertani (LB) broth amended with the antibiotics NA (20 μg/mL) and TE (5 μg/mL) followed by incubation at 37°C with shaking (150 rpm) for 7 days. A pure culture, called strain AtetA, was obtained after successive streak plates on eosin methylene blue (EMB) agar with the same antibiotics, on which it formed nucleated colonies with a metallic green sheen. Plasmids were extracted from a representative colony by alkaline lysis (Kramer and Coen, 2001) and used to transform competent *Escherichia coli* JM109 (Promega) by heat-shock according to the manufacturer’s instructions. Transformants were selected on LB agar amended with TE (10 μg/mL) and screened for the presence of plasmids by alkaline lysis and gel electrophoresis. Strain AtetA was identified by PCR amplification and 2X sequencing of the nearly complete 16S rRNA gene as previously described (Lane et al., 1985).

### Exogenous Plasmid Capture

In order to capture plasmids by the exogenous method, bacteria were gently removed from 5 g wetland sediments by incubation in 25 mL sterile Na_4_P_2_O_7_ (0.1%) for 1 h with stirring. The suspension was allowed to settle for 1 h after which 10 mL of the liquid phase was decanted. Cells were collected from the liquid phase by centrifugation at 10,000 × *g* for 10 min. and the pellet was re-suspended in 3 mL sterile saline (0.85% NaCl). Mating was initiated by mixing 0.5 mL donor bacteria suspension with 0.5 mL recipient bacteria [either *Escherichia coli* HY842 (RIF^R^, S^R^, ZEO^R^) (Brown et al., 2013) or *Pseudomonas putida* KT2440 (C^R^) (Nelson et al., 2002) (16-h culture in LB broth)] followed by immobilization onto a 0.22-μm nitrocellulose membrane filter. Control cultures were prepared identically with either donor or recipient bacteria alone. Filters were incubated overnight at 30°C on LB agar plates (no antibiotics). Biomass was removed with a sterile cotton swab and re-suspended in 1 mL sterile saline. Fifty μL of suspended bacteria, either donor, recipient, or the mated combination, was spread onto LB agar plates with (1) no antibiotics, (2) recipient-selective antibiotics alone (RIF, S, ZEO each at 100 μg/mL; C at 50 μg/mL), (3) plasmid-selective antibiotic alone (see below), or (4) recipient-selective antibiotics and plasmid-selective antibiotic combined, and incubated at 37°C for up to 4 days. Cycloheximide (100 μg/mL) was added to suppress fungal growth. Plasmid-selective antibiotics included AMP (100 μg/mL), or AMP (100 μg/mL) plus TE (10 μg/mL). Putative transconjugants were first confirmed to be the recipient strain by randomly amplified polymorphic DNA (RAPD) PCR using primers 208 and 272 (Mahenthiralingam et al., 1996) and then screened for the presence of plasmids by alkaline lysis and gel electrophoresis (Kramer and Coen, 2001).

### Mobilization Experiments

The transmissibility of each plasmid was tested with the filter-mating protocol described above using *E. coli* HY842 (RIF^R^, S^R^, ZEO^R^) or *E. coli* JM109 (NA^R^) as recipients.

### Antimicrobial Susceptibility Testing

*Escherichia coli* JM109 with and without plasmids was repeatedly (*n* = 5) subjected to antimicrobial susceptibility testing (AST) against a broad range of antibiotics by the disk diffusion method according to CLSI protocols and standards (Clinical and Laboratory Standards Institute, 2015, 2017) with disks obtained from BD or Oxoid. Minimum inhibitory concentrations (MICs) were estimated with Etests (bioMérieux). *E. coli* ATCC 25922 was tested for quality control.

### Determination of Plasmid Copy Number by Quantitative Polymerase Chain Reaction

Plasmid copy numbers were determined in *E. coli* JM109 according to the quantitative polymerase chain reaction (qPCR) method of Loftie-Eaton et al. (2014). Total cellular DNA was extracted from 5, 50, and 500 μL of early stationary phase cells with the GenElute Bacterial Genomic DNA Kit (Sigma–Aldrich) according to the manufacturer’s instructions. Chromosomal and plasmid DNA (0.04 ng/μL) was quantified in triplicate from each extraction by qPCR with primers (750 nM each) targeting either the *atpB* gene of *E. coli* (Yano et al., 2012), which exists on the chromosome in single copy, or one of the single-copy antibiotic resistance genes encoded on each plasmid: *tetA* on pLNU-11 and pTRE-1611 (Guarddon et al., 2011), *bla*_*OXA*-1_ on pTRE-131 (Knapp et al., 2010), and *bla*_*CTX-M*-55_ pTRE-2011 (Ellem et al., 2011). Amplification efficiency [*E* = 10^(-1/m)^] for all primer pairs was greater than 1.96 based on 10-fold serial dilutions of total DNA (*R*^2^ > 0.99). Gel electrophoresis and melting curve analysis of the qPCR products confirmed amplification specificity. Oligonucleotide PCR primers are described in Supplementary Table S1.

### Plasmid Genome Sequencing, Annotation, and Analysis

Total DNA was extracted using the GenElute Bacterial Genomic DNA Kit (Sigma), following the manufacturer’s instructions. Full genome sequencing was performed by the IBEST Genomic Resources Core using a MiSeq sequencer (Illumina, San Diego, CA, United States) and 250 bp Paired-End Sample Preparation Kits (Illumina, San Diego, CA, United States). Prior to analysis, duplicate read pairs were removed using a custom Python script. Sequencing adapters and low-quality bases were removed using the software package Seqyclean^1^. All remaining reads were mapped to the chromosome of either *E. coli* O157:H7 (BA000007.2) or *P. putida* KT2442 (NC_002947.3) using Bowtie2. The unused reads (i.e., non-chromosomal reads) were then assembled *de novo* with Newbler. Automated annotation was performed by the IGS Analysis Engine (University of Maryland, School of Medicine, Institute for Genome Sciences) and then curated manually. Any open reading frames shorter than 100 amino acids in length with no alignments to previously annotated sequences were not included in the final annotations. Plasmid maps were constructed using Geneious v. 8.1.3 (Biomatters Ltd.).

The genome sequence of plasmid pTRE-2011 was completed using the Pacific Biosciences Single Molecule Real Time (SMRT) sequencing platform at Washington State University’s Molecular Biology and Genomics Core. The plasmid was sequenced on a PacBio RS instrument (Pacific Biosciences, Menlo Park, CA, United States) using SMRT sequencing technology.

Plasmid sequences were compared for genomic similarity to fully sequenced plasmids published in GenBank (Benson et al., 2014). Contiguous regions of plasmid backbone genes (those involved in plasmid replication, conjugation, mating pair formation, partitioning, or stability) were used to identify other similar reference plasmids in the GenBank database using NCBI BLAST based on total BLAST alignment scores. Comparative genomic analysis of plasmid organization was performed using Mauve (Darling et al., 2010).

Identification of putative plasmid incompatibility groups, along with insertion sequences and integrons, was performed using a variety of web-based resources. Plasmid incompatibility groups were identified using PlasmidFinder 1.3 and specific plasmid multi-locus sequence type (pMLST) was identified with pMLST-1.4 (Carattoli et al., 2014). Insertion sequences were identified using ISFinder (Siguier et al., 2006), and INTEGRALL was used to identify integrons and gene cassettes (Moura et al., 2009).

Phylogenetic analysis of the amino acid sequence of the AmpC-like β-lactamase found on plasmid pLNU-11 was performed using MEGA v. 7 (Kumar et al., 2016) using MUSCLE alignments (Edgar, 2004). The best-fit evolutionary model was determined based on the minimum Bayesian Information Criterion (BIC) and Akaike Information Criterion (AIC), which were computed for all 56 amino acid evolution models in MEGA using an initial neighbor joining tree. The best fit evolutionary model was then used to identify a maximum likelihood phylogenetic tree. Reliability of the best fit tree was assessed by identifying branches conserved in other best fit maximum likelihood phylogenetic trees fit to 500 bootstrap replicates.

The sequence type of the native host organism of plasmid pTRE-2011 was determined using its complete genomic sequence (determined with the PacBio platform) and the MLST tool (Larsen et al., 2012) hosted at the Center for Genomic Epidemiology^2^.

### Nucleotide Accession Numbers

The plasmid nucleotide sequences reported in this study have been deposited in the GenBank database^3^ under the accession numbers KX863568-KX86357. The nearly complete 16S rRNA gene sequence of *Citrobacter freundii* strain AtetA has been deposited in the GenBank database under the accession number KF245926.1. The *ampC*-like gene found on plasmid pLNU-11 has been deposited in the GenBank database under the accession number KY018700 and has been assigned the allele designation *bla*_*WDC*-1_ by NCBI. The wild type host of plasmid pLNU-11, *C. freundii* strain AtetA, also carries an *ampC*-like β-lactamase gene on its chromosome whose nucleotide sequence has been deposited in the GenBank database under the accession number KX863576.

## Results

Using endogenous and exogenous capture methods, four novel antibiotic resistance plasmids were isolated from sediments of the Tijuana River Estuary (**Table 1**). The four plasmids, representing four incompatibility groups, varied in accessory genes, size, transmissibility, and copy number. The four wetland plasmids share highly conserved backbones, >97% identity, with previously described plasmids from clinical specimens and waste water treatment plants (WWTPs) around the world. However, the genomes of the wetland plasmids contain many insertions not previously associated with similar already known plasmids. Each plasmid is described below in terms of how it was captured, its transmissibility to other bacteria, the resistance phenotype it confers to an *E. coli* host, and its genetic organization.

**Table 1:** Summary of the complete genomes of four multidrug resistance plasmids captured from the Tijuana River Estuary.

Plasmid (acc. no.)^a^	Capture method (host)	Size (bp)	Inc group^b^	PCN^c^	Antibiotic resistance genes
pLNU-11 (KX863568)	Endogenous (*C. freundii* AtetA)	35,840	IncP-6	14.97 ± 3.13	*tetAR, folP, qacE*Δ*l^d^, dfrA10*, *bla*_*WDC*-1_
pTRE-1611 (KX863570)	Exogenous (*P. putida* KT2440)	45,943	IncR	2.77 ± 0.46	*sulII, tetA/R, strA/B, blaTEM-1*
pTRE-131 (KX863569)	Exogenous (*E. coli* HY842)	47,640	IncN3	238.6 ± 6.61	*aac(6’)-Ib*, *bla*_*OXA*-1_, *catB,, qacE*Δ*1d, sulI, qnrB, qacE*Δ*1d, sulI*
pTRE-2011 (KX863571)	Endogenous (*E. coli* TRE-T11)	143,600	IncFIB/FII	0.88 ± 0.12	*sulII, sulIII, floR, tetA/R, bla_CTX-M-55_, bla_TEM-1b_, aadA1, aac(3)-IIa, strA/B*


*^a^GenBank accession number. ^b^Incompatibility (Inc.) groups were determined using PlasmidFinder v 1.3 (Carattoli et al., 2014). ^c^PCN, plasmid copy number per cell (mean ± 1 *SD*, *n* = 3) (Loftie-Eaton et al., 2014). ^d^Antiseptic/biocide resistance.*

### Plasmid pLNU-11

The first plasmid, pLNU-11, was found in strain AtetA, which was selectively enriched from TRE wet-season sediments in LB broth supplemented with the antibiotics NA (20 μg/mL) and TE (5 μg/mL). A pure colony type was purified through successive passes on EMB agar, where it formed metallic green colonies typical of Gram-negative, lactose-fermenting bacteria. The 16S rRNA gene of strain AtetA shared >99% sequence similarity (1432/1435 bp) with *C. freundii* strain JCM 24061 (AB548826), warranting designation as a *C. freundii* strain. A plasmid preparation of *C. freundii* AtetA showed four distinct bands upon agarose gel electrophoresis. Additionally, a tetracycline-resistant *E. coli* JM109 transformant of this plasmid preparation possessed a plasmid with the same electrophoretic mobility as the largest of the four bands in the *C. freundii* plasmid preparation. The pLNU-11 plasmid copy number was estimated to be approximately 15 copies per cell in *E. coli* JM109 (**Table 1**).

**FIGURE 1:**
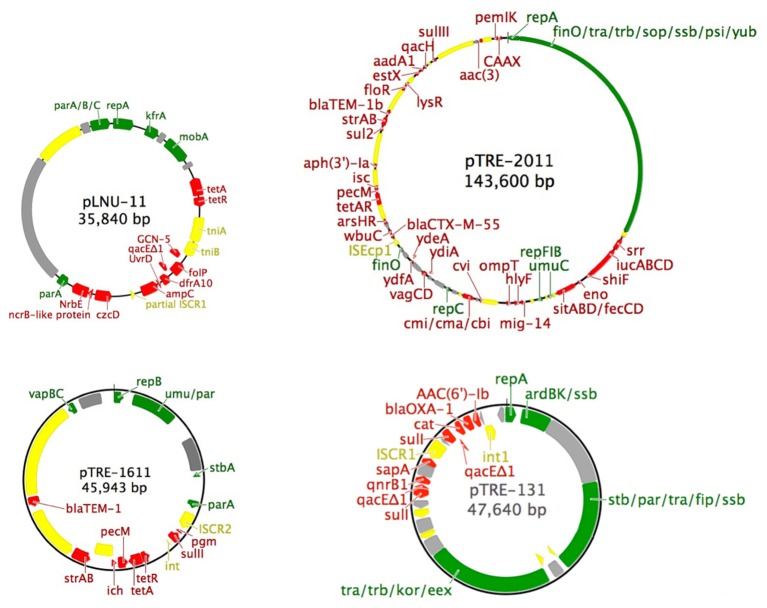
Simplified maps of the four plasmids described in this study. Color scheme: green, backbone genes (partitioning, replication, mobilization, transfer, mating pair formation and stability); gray, unidentified open reading frames; yellow, mobile genetic elements (insertion sequences, transposons, and integrons); red, accessory genes (antibiotic and heavy metal resistance). The following atypical abbreviations were used: dhps, dihydropteroate synthase; icr, isochorismatase hydrolase; pgm, phosphoglucomutase; srr, siderophore receptor.

Mating experiments between either *C. freundii* AtetA or *E. coli* JM109 (pLNU-11) as donor and *E. coli* HY842 as recipient were unsuccessful, suggesting that plasmid pLNU-11 is not self-transmissible (i.e., not conjugative). This observation was further validated by examination of the plasmid DNA sequence, which lacks any conjugative transfer genes (**Figure 1**).

The 36-kb pLNU-11 plasmid genome (KX863568) comprises a putative IncP-6 replicon, with 36 open reading frames, only six of which have been associated with essential backbone function, namely the *parA/B/C* operon, *repA*, *kfrA*, and *mobA* (**Figure 1**). Members of this incompatibility group are all mobilizable plasmids ranging in size from 13 to 57 kb. They are capable of replicating in a broad host-range extending beyond *E. coli* and *P. aeruginosa*. Relatively few IncP-6 plasmids have been fully sequenced, including Rms149 (AJ877225), pCOL-1 (KC609323), and p10265-KPC (KU578314) from clinical strains of *P. aeruginosa* (Haines et al., 2005; Naas et al., 2013; Dai et al., 2016); pRIO-5 (JF785550) from a clinical strain of *Serratia marcescens* (Bonnet et al., 2000; Bonnin et al., 2012); pRSB105 (DQ839391), a plasmid captured from a German WWTP (Schlüter et al., 2007); and pHH2-227 (JN581942), an uncultured IncW/IncP-6 hybrid plasmid that was exogenously captured from an arable soil (Heuer et al., 2009; Król et al., 2013).

The backbone regions of pLNU-11 showed high sequence similarity to multiple known plasmids of both clinical and environmental origin. The greatest similarities (>97% identity) were observed with the *S. marcescens bla*_*GES*-5_ plasmids pG5A4Y201 (KJ541069) and pG5A4Y426 (KJ541068), and the *E. coli bla*_*GES*-5_ plasmids pG5A4Y217 (KJ541071) and pG5A4Y413 (KJ541070) from nosocomial infections in Canadian hospitals (Boyd et al., 2015); Rms149, first identified in a clinical strain of *P. aeruginosa* in Germany (Haines et al., 2005); pRSB105; and the *Aeromonas hydrophila* plasmid pKPC2 (KR014106) from China (unpublished GenBank citation). Like pLNU-11, each of these similar plasmids lacks genes known to be involved in plasmid transfer, such as the *tra* or *trb* operons, implying that they are all mobilizable plasmids that require the presence of a conjugative plasmid to be transferred. Mauve alignments showed the backbone genes to be highly conserved, interrupted by accessory regions not necessarily shared by one another, and all were predicted to be mobilizable rather than self-transmissible, typical of IncP-6 plasmids (Supplementary Figure S1).

The plasmid pLNU-11 genome shows remnants of multiple insertion events, with dozens of intact or partial mobile elements dispersed throughout, similar to the genomes of other IncP-6 plasmids. For example, it possesses numerous partial and complete transposases from transposon families Tn3, IS5, IS4, and IS66. Additionally, a partial IS*CR* transposase is located between the *bla*_*WDC*-1_ and *czcD* genes, upstream of the 3′ conserved sequence of a class 1 intregron. The integron, however, is missing the 5′ conserved sequence, which would normally possess the integrase and the *attI* cassette integration site. It appears that, after integrating several cassettes, pLNU-11 lost a portion of its genome beginning within the IS*CR* transposase and continuing upstream to include the integrase and *attI* site.

Plasmid pLNU-11 contains a unique accessory region encoding predicted resistance against the tetracyclines (*tetA/R*), sulfonamides (*folP*, *dfrA10*), quaternary ammonium compounds (*qacE*Δ*1*), β-lactams (*ampC*-type β-lactamase), and aminoglycosides (*GCN-5*-like acetyl transferase). Additionally, it encodes a collection of genes predicted to be involved in heavy metal resistance [an *nrbE*-like major facilitator superfamily (MFS) protein, *czcD*, and *arsR*].

**FIGURE 2:**
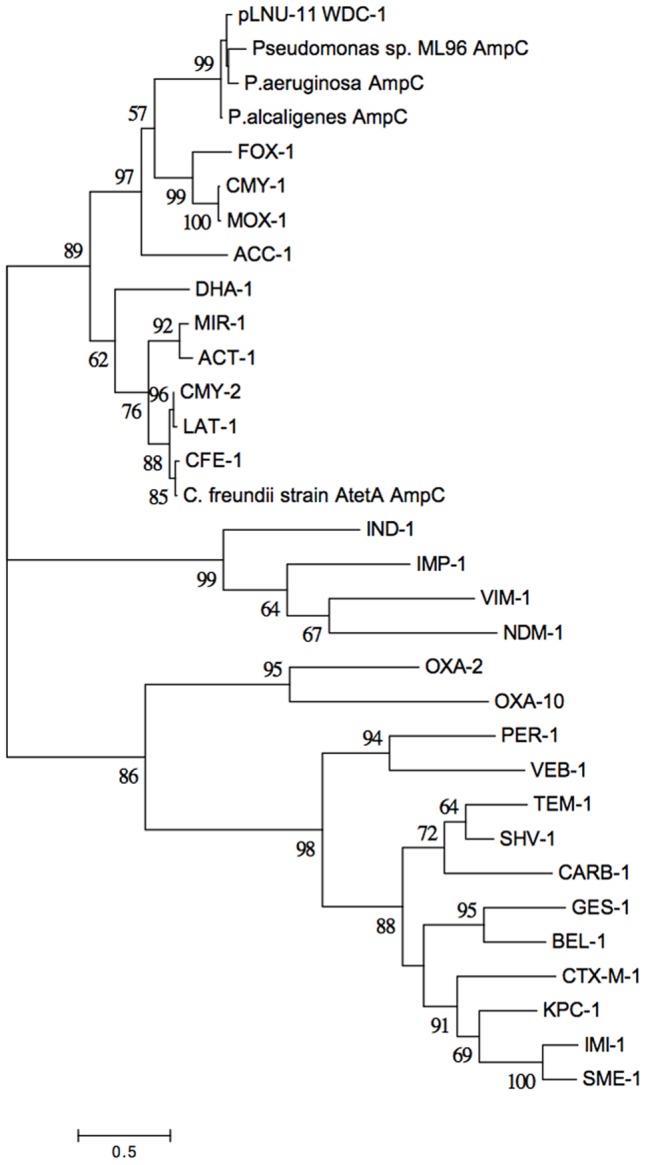
Evolutionary relationships of WDC-1 to representatives from the other classes of β-lactamases. The phylogeny was inferred from the amino acid sequences of each protein using the Maximum Likelihood method based on the Whelan and Goldman model (Whelan and Goldman, 2001). The tree with the highest log likelihood (–9267.3106) is shown. Five-hundred bootstrap replicates were used to establish support for the tree topology and the percentage of trees in which the associated taxa clustered together is shown next to the branches for all branches with over 50% support. Initial tree(s) for the heuristic search were obtained automatically by applying Neighbor-Joining and BioNJ algorithms to a matrix of pairwise distances estimated using a JTT model, and then selecting the topology with superior log likelihood value. A discrete Gamma distribution was used to model evolutionary rate differences among sites [five categories (+G, parameter = 9.5913)]. The tree is drawn to scale, with branch lengths measured in the number of substitutions per site. The analysis involved 32 amino acid sequences. All positions with less than 95% site coverage were eliminated. There was a total of 185 positions in the final dataset. Evolutionary analyses were conducted in MEGA7 (Kumar et al., 2016).

A novel AmpC-like β-lactamase was discovered on pLNU-11 upstream of *uvrD*, *dfrA10*, *qacE*Δ*1*, *folP*, and *GCN-5* (**Figure 1**). The novel allele shares less than 62% amino acid sequence identity with other characterized enzymes, namely those from the PDC class of β-lactamase found in *P. aeruginosa*. The new AmpC-like β-lactamase on pLNU-11 has been designated WDC-1 (wetland-derived class C β-lactamase) by the NCBI. It shares high amino acid sequence identity (>99% coverage and >93% identity) to three uncharacterized AmpC-like proteins in GenBank (WP_061904073.1, WP_021699607.1, and WP_025297965.1m). In fact, there are over 100 entries of this protein in GenBank, however, all are located in chromosomal sequences from different species of *Pseudomonas*. Together, these four proteins form a distinct sub-class of β-lactamases (**Figure 2**). The *bla*_*WDC*-1_ gene on pLNU-11 appears to be the first allele of this protein found on a plasmid. Disk diffusion and Etest results indicate that WDC-1 is primarily an oxacillinase and cephalothinase (**Table 2** and Supplementary Table S2). Note that, like many *Citrobacter* spp., *C. freundii* AtetA carries a chromosomal *bla_AmpC_* allele distinct from *bla*_*WDC*-1_ found on pLNU-11 (“*C. freundii* strain AtetA AmpC” in **Figure 2**).

**Table 2:** Antibiotic resistance profiles^a^ of *E. coli* JM109 with four environmental plasmids and of the two original host strains described in this study.

Plasmid	Clinically resistant^b^	Decreased susceptibility^c^
pLNU-11	CF, D, TE	AM, SAM, SXT
pTRE-1611	AM, PIP, S, TE, TIC	SAM, SXT
pTRE-131	AM, TIC	AN, C, CIP, FEP, GAT, K, LOM, LVX, MXF, NOR, OFX, PIP, SAM, SPX, TIM, TZP
pTRE-2011	AM, ATM, C, CF, CIP, CTX, D, GAT, K, LOM, LVX, MXF, NOR, OFX, PIP, SAM, SXT, TE, TIC	CL, FEP, TIM
*C. freundii* AtetA^d^	AM, **CF**, **D**, LOM, MXF, NA, S, SXT, **TE**	
*E. coli* TRE-T11^e^	AN, **ATM**, CAZ, **CF**, **CIP**, CL, **CTX**, CXM, **D**, FOX, **GAT**, **K**, **LOM**, **LVX**, **MXF**, **NOR**, **OFX**, **PIP**, S, SPX, **SXT**, **TE**, **TIC**	


*^a^Antibiotic abbreviations are defined in the section “Materials and Methods”. ^b^Clinical resistance is defined as a zone of inhibition smaller than the resistance breakpoints set for the *Enterobacteriaceae* by the CLSI (Clinical and Laboratory Standards Institute, 2015, 2017). ^c^Decreased susceptibility is defined here as a statistically significant decrease of ≥3 mm (*n* = 5, two-tailed *t*-test assuming unequal variance, *p* < 0.01) in zone of inhibition relative to *E. coli* JM109 without plasmid. ^d^*C. freundii* strain AtetA is the original host of plasmid pLNU-11. Resistances conferred to *E. coli* JM109 by pLNU-11 are in boldface type for reference. ^e^*E. coli* strain TRE-T11 is the original host of plasmid pTRE-2011. Resistances conferred to *E. coli* JM109 by pTRE-2011 are in boldface type for reference.*

The original host of pLNU-11, *C. freundii* AtetA, was resistant (as defined by CLSI breakpoints) to β-lactams (AM, CF), tetracyclines (D, TE), (fluoro)quinolones (LOM, MXF, NA), an aminoglycoside (S), and sulfonamide (SXT) (**Table 2**), and showed intermediate resistance to three other (fluoro)quinolones (CIP, GAT, OFX) and a β-lactam (FOX) (intermediate phenotypes not shown in **Table 2**). The transformant *E. coli* JM109 (pLNU-11) showed resistance to CF, D, and TE, though decreased susceptibility relative to *E. coli* JM109 without plasmid was also observed to AM, SAM, and SXT (**Table 2**). The resistance to (fluoro)quinolones or S observed in *C. freundii* AtetA could not be explained by pLNU-11, suggesting that resistance to these antibiotics may have been chromosomally encoded or located on one of the smaller plasmids. Plasmid-mediated resistance to NA cannot be tested in *E. coli* JM109 since it is naturally resistant to NA. Although pLNU-11 encodes a *GCN-5*-like acetyltransferase, no S resistance was observed in the JM109 transformant, suggesting that the gene might not be functional.

### Plasmid pTRE-1611

Plasmid pTRE-1611 was captured from wetland bacteria into *P. putida* KT2440 as recipient under selection for TE resistance. As *P. putida* KT2440 is broadly resistant to antibiotics, we attempted to transfer pTRE-1611 to *E. coli* HY842 by mating, but were unsuccessful, suggesting that it is not self-transmissible. The plasmid was transferred by heat-shock transformation to *E. coli* JM109 where resistance profiles could be more readily studied. Additional mating experiments between *E. coli* JM109 (pTRE-1611) and *E. coli* HY842 were also unsuccessful. Although, pTRE-1611 was originally captured by conjugation, this behavior was not demonstrated again, suggesting that it was likely mobilized into *P. putida* KT2440 by a self-transmissible plasmid that was either not co-transferred or was subsequently lost from the recipient. Plasmid pTRE-1611 was maintained in *E. coli* JM109 at approximately 3 copies per cell (**Table 1**).

Plasmid pTRE-1611 conferred resistance to *E. coli* JM109 against three β-lactam drugs (AM, PIP, TIC), a tetracycline (TE), and an aminoglycoside (S) (**Table 2**). Decreased susceptibilities to SAM and SXT were also noted, though resistant breakpoints were not surpassed.

The 46-kb pTRE-1611 genome (KX863570) has a putative IncR replicon and 39 open reading frames. Although IncR plasmid replicons were first described in *Salmonella* strains (García-Fernández et al., 2009), they have since been more commonly found in *Klebsiella pneumoniae* (e.g., Compain et al., 2014), and are often associated with the epidemic metallo-β-lactamase NDM-1 (e.g., Kocsis et al., 2016). IncR replicons are characteristically non-conjugative. Plasmid pTRE-1611 does not carry *bla*_*NDM*-1_.

The simple backbone of plasmid pTRE-1611 consists of the *repB, parA/B, umuC/D*, and *stbA* genes and is absent of any *tra* or *trb* genes necessary for conjugation. Additionally, it encodes the *vapB/C* type II toxin-antitoxin system, along with phosphoglucomutase, *pecM*, and an isochorismatase-like hydrolase, each of which has been implicated in resistance or virulence (Ugalde et al., 2000; Maruyama and Hamano, 2009; Burska and Fletcher, 2014). Antibiotic resistance genes include *sulII, tetA/R, strA/B*, and *bla*_*TEM*-1_. Each of the resistances and decreased susceptibilities observed in *E. coli* JM109 (**Table 2**) could be accounted for with these four resistance genes or pairs of genes.

Like plasmid pLNU-11, pTRE-1611 is littered with several diverse transposons from the families Tn3, IS3, IS110, IS91, IS5, IS6, and IS1, some partial and others complete. A small portion of a class 1 integrase is present downstream of *sulII* along with what appears to be an IS*CR2* element (Poirel et al., 2009) located upstream of *sulII* (**Figure 1**). It is not uncommon for IS*CR2* elements to be associated with a 3′ conserved sequence of an integron, including *sulII*, but lacking the 5′ conserved sequence (Toleman et al., 2006).

The small backbone regions of pTRE-1611 only shared similarity (>85% coverage, >98% identity) with the *K. pneumoniae* plasmids pKP1034 (KP893385), pK245 (DQ449578), pKPN5 (CP000650), and pWSZBR (CP015991), all of which lack *tra/trb* operons and thus would be predicted to be incapable of self-transmission. Of these plasmids, only pK245 could be tracked to a source, a clinic in Tainan, Taiwan (Chen et al., 2006). The others were only recently submitted to GenBank (2015 or later) and had not been published as of May 2017. Mauve alignment of pTRE-1611 with the above four plasmids highlights the similar backbones with distinct accessory regions that include *tetA/R*, *pecM*, and *bla*_*TEM*-1_ (Supplementary Figure S2).

### Plasmid pTRE-131

The third plasmid, pTRE-131, was also captured from wetland bacteria, but now into *E. coli* HY842 as recipient with selection for AM resistance. pTRE-131 was readily transferred back and forth between *E. coli* HY842 and *E. coli* JM109 by conjugation, establishing it as a self-transmissible plasmid. Remarkably, the 48-kb plasmid was apparently maintained at approximately 240 copies per *E. coli* JM109 cell (**Table 1**). This unusually high copy number was confirmed repeatedly, and in line with a much stronger plasmid band on agarose gels compared to the other plasmids. Whereas the high copy number may be imposing a high cost to its bacterial host, it very likely also affords the plasmid greater opportunity to persist within a population absent of a selective pressure.

*Escherichia coli* JM109 (pTRE-131) displayed resistance to only AM and TIC (**Table 2**), but susceptibility was decreased to many other classes of antibiotics including fluoroquinolones, chloramphenicol, aminoglycosides, and sulfonamides, qualifying it for multi-drug resistance status.

The pTRE-131 plasmid genome (KX863569) has a putative IncN3 broad-host-range replicon (Götz et al., 1996) with 55 open reading frames. The backbone comprises *repA, stbA/C, parA, ardB/K, ssb, traA/B/C/D/E/F/G/I/K/L/O, fip, trbC, eex, korA/B, trwB*, typical of IncN replicons (**Figure 1**). The backbone regions of pTRE-131 share high similarity (>93% identity, >95% coverage) with multiple plasmids in GenBank including the *K. pneumoniae* plasmids p0801-IMP (KT345947), pJIE137 (EF219134), pKPC-SMH (KT148595) and pTR3 (JQ349086), the *Enterobacter hormaechei* plasmids p34998-53 (KT148595) and p34983-59.134kb (CP010378), and the *E. coli* plasmids pACT2-NDM-1 (KP826703), pEC448_OXA163 (CP010382), pNDM-ECS01 (KJ413946) and p271A (JF785549). All other alignments to this backbone produced less than 65% coverage during alignment.

The pTRE-131 genome contains several partial insertion sequences, a complete Tn3-like transposon, and a complex IS*CR1* element embedded within a class 1 integron (**Figure 1**). The integron begins in the 5′ conserved region with an integrase, followed by five resistance genes: *aac(6*′*)-Ib*, *bla*_*OXA*-1_, *catB*, *qacE*Δ*1*, and *sulI*. The last two genes in the list, *qacE*Δ*1* and *sulI*, typically form the 3′ conserved sequence of the integron. However, in the case of pTRE-131, the first *sulI* gene is followed by an IS*CR1* element and four more accessory genes: *sapA*, *qnrB*, *qacE*Δ*1*, and *sulI*, forming the 3′ conserved region and ending the integron. The *sapA*-like gene has been implicated as a virulence factor involved in resistance to host antimicrobial peptides (Parra-Lopez et al., 1993), while the others confer resistance to aminoglycosides (*aac(6*′*)-Ib*), β-lactams (*bla*_*OXA*-1_), chloramphenicol (*catB*), disinfectants (*qacE*Δ*1*), sulfonamides (*sulI*), and (fluoro)quinolones (*qnrB*). The resistance phenotype of *E. coli* JM109 (pTRE-131) (**Table 2**) indicates that each of the drug resistance genes in the genome (**Table 1** and **Figure 1**) is functional.

Mauve alignments with the top five best aligning backbones show that only pJIE137 carries the complex IS*CR1* element present in pTRE-131 (Supplementary Figure S3), however, the *cat*, *bla*_*OXA*-1_ and *aac(6’)-Ib* genes are not present in pJIE137. Instead, pJIE137 carries a *dfrA12*, followed by an unknown protein and *aadA2* located in the same position. Of the database entries annotated with source information, this backbone appears to be globally dispersed with examples from Australia (Partridge et al., 2012), Thailand (Netikul et al., 2014), eastern Canada (Tijet et al., 2016), and Singapore (Chen et al., 2012). This report now adds the southwestern United States to its expanding distribution map.

### Plasmid pTRE-2011

During a bi-parental mating experiment with wetland bacteria as plasmid donor, a homogeneous colony type appeared on a control plate containing RIF, S, ZEO, and CTX that was inoculated with only environmental bacteria. The pure culture, named strain TRE-T11, contained two high-molecular-weight plasmids. The first, pTRE-2011, was easily transferred to *E. coli* HY842 or *E. coli* JM109 by mating with CTX, where it was maintained at an average of fewer than one copy per cell (**Table 1**). Strain TRE-T11 produced metallic green colonies on EMB agar, and biochemical evidence using the API 20E rapid identification kit (bioMérieux) indicated that it is a strain of *E. coli* (data not shown). The second plasmid was sequenced with the PacBio platform and found to carry no antibiotic resistance genes or other accessories. It was therefore not studied further.

The genome of strain TRE-T11 was sequenced using PacBio and identified as the broadly distributed *E. coli* ST744 by multi-locus sequence typing. ST744 distribution includes Europe (Guenther et al., 2012; Brolund et al., 2014; Wagner et al., 2014; Hasman et al., 2015; Loncaric et al., 2016), South(east) Asia (Hasan et al., 2012; Stoesser et al., 2012; Cai et al., 2014; Ho et al., 2016), Australia (Sidjabat et al., 2014; Abraham et al., 2015), and North Africa (Belmahdi et al., 2016). To our knowledge, this is the first report of ST744 in the Americas.

*Escherichia coli* TRE-T11 demonstrated resistance to no fewer than 23 of the antibiotics tested, including aminoglycosides (AN, K, and S), a monobactam (ATM), β-lactams (CAZ, CF, CTX, CXM, FOX, PIP, and TIC), fluoroquinolones (CIP, GAT, LOM, LVX, MXF, NOR, OFX, and SPX), a polymyxin (CL), tetracyclines (D and TE), and a sulfonamide (SXT) (**Table 2**). Sixteen of these resistance traits transferred to *E. coli* JM109 after conjugation (**Table 2**), including some of the β-lactams (CF, CTX, PIP, and TIC), the monobactam (ATM), most of the fluoroquinolones (CIP, GAT, LOM, LVX, MXF, NOR, and OFX), the two tetracyclines (D and TE), one of the aminoglycosides (K), and the sulfonamide (SXT). *E. coli* JM109 (pTRE-2011) was also resistant to ampicillin with and without the β-lactamase inhibitor sulbactam (AM and SAM) as well as chloramphenicol (C), neither of which was detected in the original *E. coli* TRE-T11 host (**Table 2**). Furthermore, a statistically significant decrease in zone of inhibition was observed in *E. coli* JM109 (pTRE-2011) for the antibiotics CL, FEP, and TIM, although CLSI clinical resistance breakpoints were not achieved (**Table 2**). Much, but not all, of the resistance phenotype of *E. coli* TRE-T11 could be transferred with plasmid pTRE-2011.

The 144-kb pTRE-2011 plasmid genome (KX863571) (**Figure 1**) consists of 160 open reading frames including a putative IncFIB/FII multi-replicon backbone (ST F18:A-:B1 according to the pMLST method of Carattoli et al., 2014) containing *sopA/B*, *ssbF*, 24 *tra* genes, nine *trb* genes, *finO*, multiple *rep* genes, and *umuD*. The backbone is highly conserved with other known plasmids. The largest contiguous backbone region shares >99% identity (100% coverage) with the globally distributed *E. coli* plasmids pMR0516mcr (KX276657), p3PCN033 (CP006635), and pCERC3 (KR827684), identified in the United States, China, and Australia, respectively (Liu et al., 2015; McGann et al., 2016; Moran et al., 2016).

Plasmid pTRE-2011 conferred resistance to an extensive list of antibiotics in *E. coli* JM109 (**Table 2**). Identifiable resistance genes (**Table 1**) are predicted to confer resistance to β-lactams, tetracyclines, aminoglycosides, sulfonamides, and amphenicols. Transferred resistance to fluoroquinolones and decreased sensitivity to colistin, however, could not be readily explained by the plasmid genome.

The *bla*_*CTX-M*-55_ gene of plasmid pTRE-2011 is associated with an IS*Ecp-1* insertion sequence, common among the *bla_CTX-M_* genes (Karim et al., 2001; Rossolini et al., 2007). IS*Ecp1* provides a highly active *Enterobacteriaceae* promoter within its 3′ sequence (Karim et al., 2001), leading to overexpression of downstream genes (Wang et al., 2013). Facile transposition of *bla_CTX-M_* genes by IS*Ecp1* between bacterial chromosomes and plasmids, including across genus boundaries (Lartigue et al., 2006; Mahrouki et al., 2012), may account for their epidemic global spread in recent decades.

Several toxin-antitoxin systems were identified including the *hok*/*sok* antitoxin (*hok* toxin was not observed), *pemI/K*, *vapB/C*, and *cmi/a*. These may account for the plasmid’s persistence despite such low copy numbers. Virulence genes were also discovered in the pTRE-2011 genome sequence including an iron acquisition system consisting of the *sitA/B/D* genes, *fecC/D*, *enolase*, *shiF*, *lucA/C/D*, and *srr*; the protease gene *ompT*; the hemolysin gene *hlyF*; and the putative virulence gene *mig-14*.

Mauve alignments with pTRE-2011 and the most similar plasmids (based on backbone similarity) in the GenBank database demonstrated that the largest contiguous region of backbone genes of pTRE-2011 were present in at least one of the other known reference plasmids. The large contiguous backbone of pTRE-2011 showed 100% identity to that of pMR0516mcr (Supplementary Figure S4), a 226-kb IncF plasmid from an *E. coli* urinary tract pathogen (McGann et al., 2016). The DNA sequence of pMR0516mcr appears to have two separate backbone regions, only one of which aligns well with plasmid pTRE-2011 described here, suggesting that pMR0516mcr is the result of a pTRE-2011-like plasmid combining with another distinct replicon to create a large, multi-replicon genome. Although the pTRE-2011 genome does not include an *mcr-1*-like colistin resistance gene, as seen in pMR0516mcr, the original host of pTRE-2011, *E. coli* TRE-T11, was indeed resistant to colistin and the transconjugant *E. coli* JM109 (pTRE-2011) showed decreased susceptibility to colistin (**Table 2**), suggesting that there may be an alternative mechanism such as a generalized efflux pump that has not been recognized.

## Discussion

Four novel multidrug-resistance plasmids, ranging in size from <36 to >143 kb, were captured from sediments of a coastal wetland during the rainy season and characterized both genetically and phenotypically. Two were captured endogenously, by first isolating the native host strain and then moving the plasmid to a lab strain for analyses, while the other two were captured by mating wetland bacteria with either *P. putida* KT2440 or *E. coli* HY842 under selective pressure. Two of the plasmids, pTRE-131 and pTRE-2011, were self-transmissible as evidenced by conjugation between *E. coli* strains. The other two plasmids, pLNU-11 and pTRE-1611, were likely mobilizable but not self-transmissible.

The four plasmids putatively belong to different incompatibility groups: IncP-6, IncN3, IncR, and IncFIB/FII. The IncP-6 and IncN3 replicons have a broad host-range (Tardif and Grant, 1982; Haines et al., 2005; Eikmeyer et al., 2012) that may allow plasmids pLNU-11 and pTRE-131 to disseminate widely in the wetlands, possibly spreading to a variety of indigenous and exogenous strains. To the best of our knowledge, IncR plasmids have only been observed in *Enterobacteriaceae* (e.g., Hordijk et al., 2011; Drieux et al., 2013; Compain et al., 2014; García et al., 2014; Du et al., 2016; Guo et al., 2016). Similarly, IncF replicons are thought to be limited to this family (Carattoli, 2009). *Enterobacteriaceae* are expected to be less abundant in natural marine wetlands during the dry season. Thus, we might predict plasmids pTRE-1611 and pTRE-2011 to spread less often and only among enteric bacteria that are introduced to the wetlands with storm water. It is noteworthy, however, that the IncR plasmid pTRE-1611 was captured and maintained initially in *P. putida* KT2440, a member of the non-enteric gamma-*Proteobacteria.* It may be that, although IncR plasmids in the literature have only been reported in species of the *Enterobacteriaceae*, they have the potential to spread to a broader host range than previously thought. Rigorous host-range studies for the various IncR plasmids are warranted to improve our understanding of this important group.

Fluoroquinolone antibiotics are among the most commonly prescribed in the world today, and, not surprisingly, resistance is on the rise (Spellberg and Doi, 2015). We recently demonstrated that various fluoroquinolone resistance genes [*qnrA*, *qnrB*, *qnrS*, *qepA*, and *aac(6’)-Ib-cr*] are present in the Tijuana River Estuary sediments in both the dry and rainy seasons, although their abundances increase with the influx of storm water (Cummings et al., 2011). In our 2011 study, *qnrA* genes were present in densities as high as 3.08 × 10^6^ copies per g sediment (equivalent to 2.10 × 10^-2^ copies per 16S rRNA gene). In the current study, the high copy-number plasmid pTRE-131 harbors a *qnrB* gene that appears to be functional based on its resistance profile. The combination of a high copy-number, broad host-range plasmid and the *qnrB* gene’s location within an intact IS*CR1* element makes for the ideal situation to broadly disseminate the gene in the TRE wetlands.

Similarly, late-generation cephalosporins are critically important in the global fight against multidrug-resistant Gram-negative pathogens, and yet cephalosporinases are spreading rapidly, threatening to undermine our ability to make the best use of these life-saving drugs. In recent years, extended-spectrum β-lactamases (ESBLs) in the CTX-M class have become commonplace. This renders the cephalosporins useless (Pitout and Laupland, 2008; Sheng et al., 2013) and forces clinicians to use less desirable alternatives such as carbapenems or polymyxins (Papp-Wallace et al., 2011). In an earlier study, we discovered *bla_CTX-M_* genes from phylogenetic groups 1 and 2 in local WWTPs, and group 1 *bla_CTX-M_* genes in the TRE sediments during the rainy season (Borgogna et al., 2016). Although their peak abundance (4.39 × 10^-5^ copies per 16S rRNA gene) was lower than that seen for *qnrA* (2.10 × 10^-2^ copies per 16S rRNA gene), their diversity was tremendous. Of 165 distinct *bla_CTX-M_* group 1 clones, 157 were novel, not matching any of the alleles deposited in the GenBank database. In that 2016 study, variants of the *bla*_*CTX-M*-3_, *bla*_*CTX-M*-30_, and *bla*_*CTX-M*-36_ alleles were most common, and no clones of the globally ubiquitous *bla*_*CTX-M*-1_ or *bla*_*CTX-M*-15_ were recovered. In the current study, plasmid pTRE-2011 harbors a group 1 *bla*_*CTX-M*-55_ gene (Kiratisin et al., 2007), which was not seen in our earlier PCR-based study, suggesting that the diversity present in the wetlands is even greater than previously estimated.

An unanswered question regarding clinically relevant plasmids in natural environments has to do with the mechanisms that allow them to persist. Certainly, if the concentrations of antibiotics in the environment are high enough, they can pose a selective pressure for plasmids carrying genes conferring resistance to those antibiotics, but this scenario is unlikely in most natural soil and water ecosystems. Our plasmids seem to each have a different putative alternative mechanism to maintain themselves. One alternative mechanism for persistence is for a plasmid to acquire other accessory genes that provide some competitive advantage to the host bacteria, such as catabolic pathways or heavy metal resistance as seen in pLNU-11. Another possible mechanism for maintaining resistance plasmids in natural ecosystems is post-segregational killing by means of toxin-antitoxin (TA) systems (Hayes, 2003), such as those located on pTRE-1611 and pTRE-2011. In a typical TA system, a stable protein toxin is produced that has some killing or inhibitory effect, such as cell membrane depolarization. At the same time, a short-lived antitoxin, either in the form of a protein that binds to and inhibits the toxin protein or an antisense RNA that inhibits toxin mRNA translation, is produced to protect the host cell. If a daughter cell does not receive a copy of the plasmid during cell division, the antitoxin is degraded leaving an active toxin to kill the cell. Such TA systems make it nearly impossible for a strain to lose its plasmid once it has acquired it. Finally, as observed with pTRE-131, maintaining a very high copy number within the cell can aid in plasmid maintenance because of the vanishingly small probability that a daughter cell would not inherit at least one copy of the plasmid if partitioning and segregation systems are functioning normally (Summers and Sherratt, 1985). These different persistence mechanisms may provide targets for productive research into ways to cure bacterial pathogens of their plasmids *in vivo* (Loftie-Eaton et al., 2016).

While chromosomal mutations may arise spontaneously and rapidly, providing an intrinsic form of antibiotic resistance that is restricted to a particular clonal population, the acquired resistance genes that can be horizontally transferred across phylogenetic boundaries within a single generation are most often modified forms of proteins with other original functions in the host. This sort of resistance mechanism does not appear quickly, and could not be blamed on the sudden and heavy use of antibiotics to treat infections over the past 70 years. It has been argued that such acquired resistance elements have evolved in natural soil and water habitats (Aminov, 2009; Martínez, 2009), and thus it is there that we should look to gain insight into both the past of acquired resistance genes as well as their possible future. The release of antibiotics, antibiotic-resistant bacteria, and antibiotic resistance genes into natural ecosystems where native bacteria carry their own mobile genome, complete with transposons, integrons, and insertion sequences, is creating the perfect storm for the recombination of genetic information into new mobile elements that may find their way back into the clinical environment. A complete understanding of resistance plasmids and the threat they pose to human health must therefore include sustained investigation into the role of the natural environment in the emergence of novel plasmids and thus novel pathogens.

## Author Contributions

RB was the main author of the text and oversaw much of the genomic analysis of the four plasmids. DC oversaw all aspects of the projects and experimental designs for all laboratory experiments. Additionally, he was a major contributor in writing the text. ET and CB were valuable consultants in designing protocols for plasmid experiments, e.g., transfer experiments and genomic analysis, e.g., phylogenetic analysis. Undergraduate students BA, CW, KD, RE, MG, VG, VH, MM, CL, JM, KM, and CV performed all of the laboratory experiments characterizing these four plasmids as part of their summer research.

## Conflict of Interest Statement

The authors declare that the research was conducted in the absence of any commercial or financial relationships that could be construed as a potential conflict of interest.

## Footnotes

1 https://github.com/ibest/seqyclean

2 www.genomicepidemiology.org

3 http://www.ncbi.nlm.nih.gov/genbank
