# Supplementary material for: Characterization of Four Multidrug Resistance Plasmids Captured from the Sediments of an Urban Coastal Wetland
Source: Front Microbiol. 2017 Oct 10;8:1922. doi: 10.3389/fmicb.2017.01922 (PMC5641379; doi:10.3389/fmicb.2017.01922)
Supplement: Supplementary file 1 [file Data_Sheet_1.PDF]

SUPPLEMENTARY MATERIALS

Genomic and Functional Analysis of Four Multidrug Resistance Plasmids Captured from the Sediments of an Urban Coastal Wetland

Ryan T. Botts[1,*], Brooke A. Apffel[2], C. Joy Walters[2], Kelly E. Davidson[2], Ryan S. Echols[2], Michael R. Geiger[2], Victoria L. Guzman[2], Victoria S. Haase[2], Michal A. Montana[2], Chip A. La Chat[2], Jenna A. Mielke[2,a], Kelly L. Mullen[2,b], Cierra C. Virtue[2], Celeste J. Brown[3], Eva M. Top[3], and David E. Cummings[2]

[1]Department of Mathematics, Information, and Computer Sciences, Point Loma Nazarene University, San Diego, CA, USA
[2]Department of Biology, Point Loma Nazarene University, San Diego, CA, USA
[3]Department of Biological Sciences and Institute for Bioinformatics and Evolutionary Studies (IBEST), University of Idaho, Moscow, ID, USA

*Correspondence:
Ryan T. Botts
3900 Lomaland Dr.
Department of Mathematics, Information, and Computer Sciences
Point Loma Nazarene University
San Diego, CA 92106, USA
ryanbotts@pointloma.edu

[a]Current address: University of California San Diego, School of Medicine, Department of Pediatrics, San Diego, CA, USA
[b]Current address: Fort Wayne Medical Education Program, Indianapolis, IN, USA

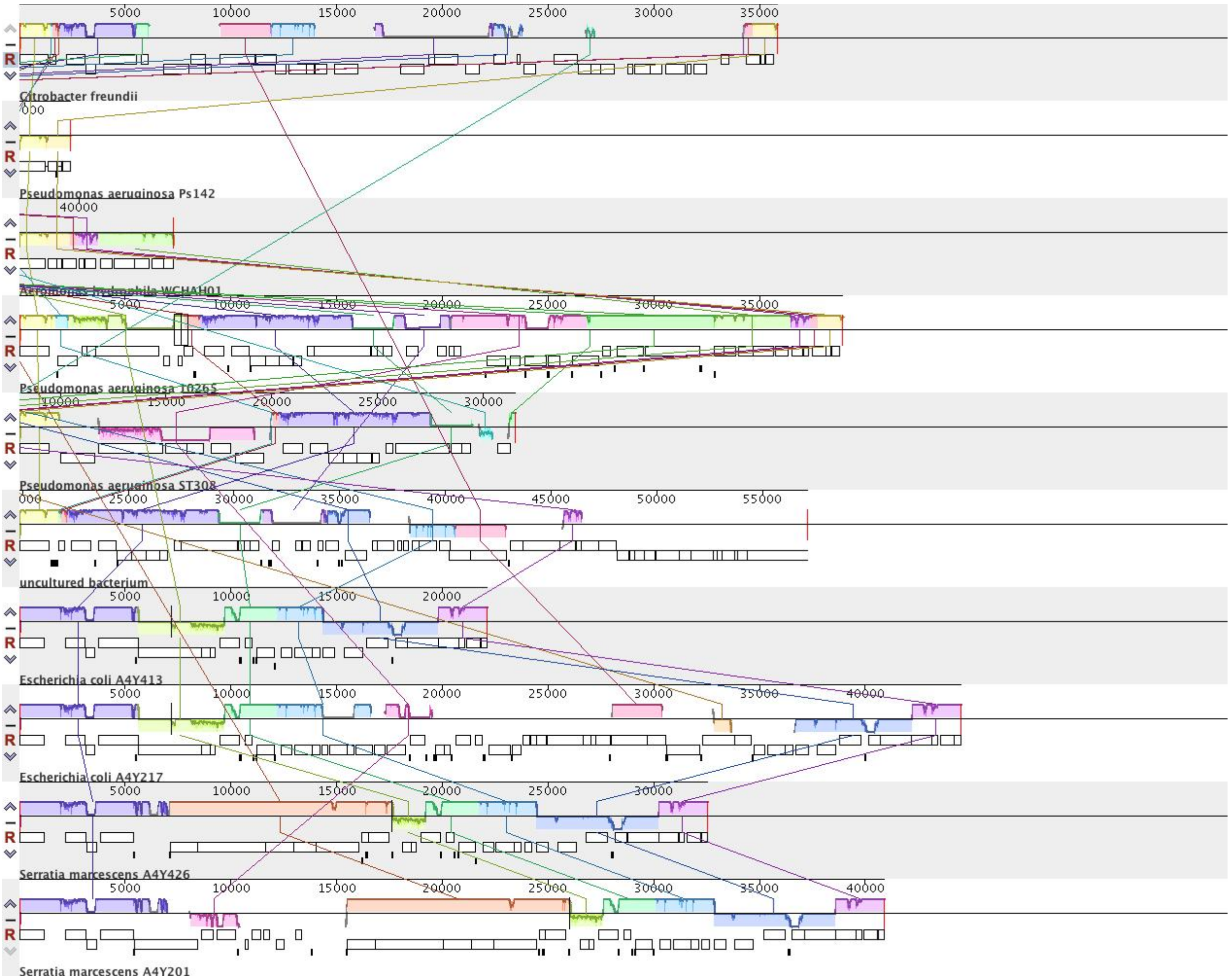

Supplemental Figure S1. MAUVE alignment of plasmid pLNU-11 and reference plasmids with most similar backbones from GenBank. Identically colored shading connected by lines indicates conserved regions.

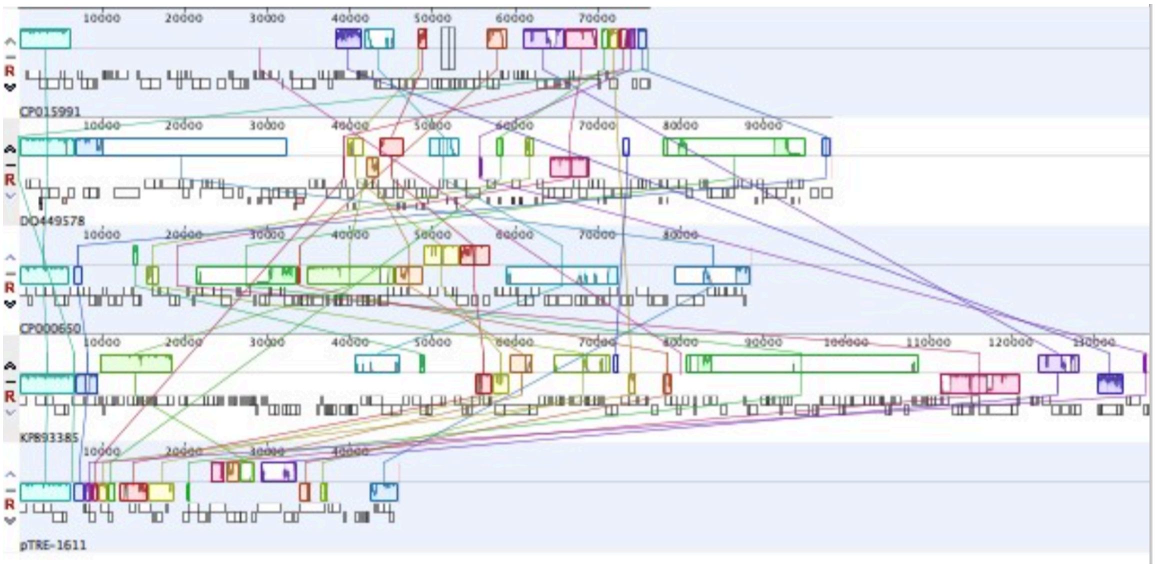

Supplemental Figure S2. MAUVE alignment of plasmid pTRE-1611 and reference plasmids with most similar backbones from GenBank. Identically colored shading connected by lines indicates conserved regions.

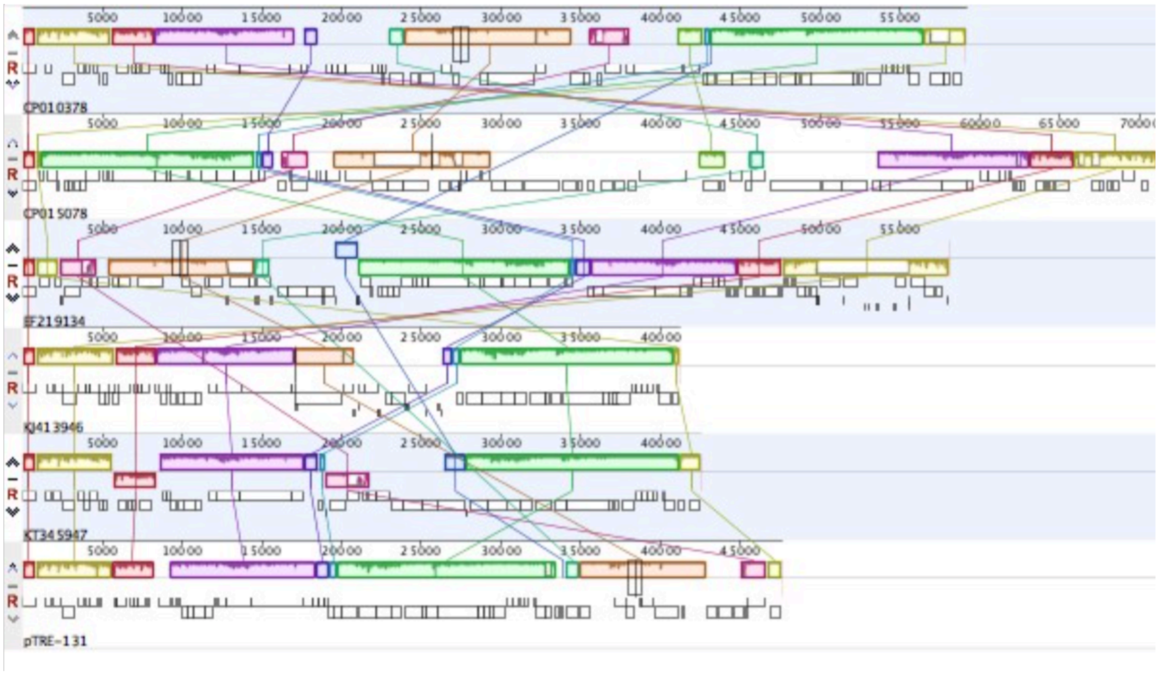

Supplemental Figure S3. MAUVE alignment of plasmid pTRE-131 and reference plasmids with most similar backbones from GenBank. Identically colored shading connected by lines indicates conserved regions.

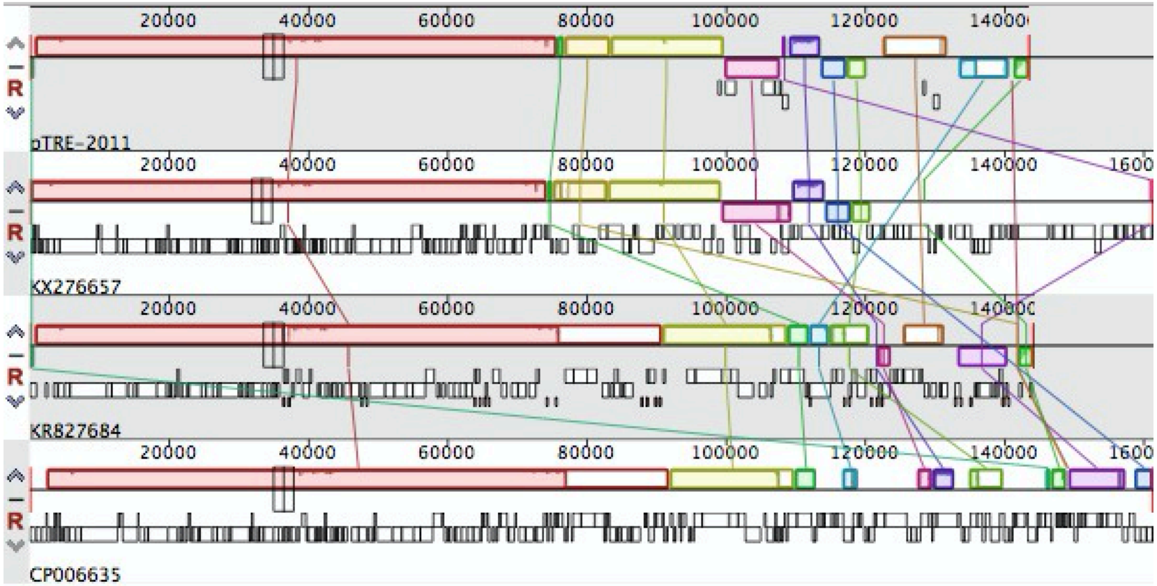

Supplemental Figure S4. MAUVE alignment of plasmid pTRE-2011 and reference plasmids with most similar backbones from GenBank. Identically colored shading connected by lines indicates conserved regions.

Supplemental Table S1. Salient features of PCR primers used in this study.

Primer	Target	Sequence (5'→3')	Amplicon size (bp)	Reference
EcoliatpF	*atpB*	GTCGGTCCAGGTCTTCATTT	157	Yano et al. 2012
EcoliatpR	*atpB*	TGCACACGGTAATCTGGAAT		
tetA-F	*tetA*	CCGCGCTTTGGGTCATT	51	Guarddon et al. 2011
tetA-R	*tetA*	TGGTCGCGTCCCAGTGA		
OXA1B14	$bla_{OXA\text{-}1}$	CACTTACAGGAAACTTGGGGTCG	79	Knapp et al. 2010
blaOXA1-R	$bla_{OXA\text{-}1}$	AGTGTGTTTAGAATGGTGATC		
CTX-M Gp1.F	$bla_{CTX\text{-}M\text{-}55}$	GGAATCTGACGCTGGGTAAA	232	Ellem et al. 2011
CTX-M Gp1.R	$bla_{CTX\text{-}M\text{-}55}$	GGTTGAGGCTGGGTGAAGTA		

References

Ellem, J., Partridge, S. R., and Iredell, J. R. (2011) Efficient direct extended-spectrum ß-lactamase detection by multiplex real-time PCR: accurate assignment of phenotypes by use of a limited set of genetic markers. *J. Clin. Microbiol.* 49, 3074-3077.

Guarddon, M., Miranda, J. M., Rodríguez, J. A., Vázquez, B. I., Cepeda, A., and Franco, C. M. (2011) Real-time polymerase chain reaction for the quantitative detection of *tetA* and *tetB* bacterial tetracycline resistance genes in food. *Int. J. Food Microbiol.* 146, 284-289.

Knapp, C. W., Dolfing, J., Ehlert, P. A. I., and Graham, D. W. (2010) Evidence of increasing antibiotic resistance gene abundances in archived soils since 1940. *Environ. Sci. and Technol.* 44, 580-587.

Yano, H., Deckert, G. E., Rogers, L. M., and Top, E. M. (2012) Roles of long and short replication initiation proteins in the fate of IncP-1 plasmids. *J. Bacteriol.* 194, 1533-1543.

Supplemental Table S2. Minimum inhibitory concentrations (MICs) of β-lactam antibiotics for *E. coli* JM109 with plasmid pLNU-11 carrying the novel Amp-C β-lactamase WDC-1.

Antibiotic	MIC (μg mL^{-1})
Ampicillin	8
Aztreonam	0.094
Cefepime	≤0.025
Cefepime-clavulanic acid	≤0.064
Cefotaxime	≤0.25
Cefotaxime-clavulanic acid	0.023
Cefoxitin	12
Ceftazidime	≤0.5
Ceftazidime-clavulanic acid	≤0.064
Cephalothin	32
Imipenem	0.125
Oxacillin	>256
